# Incidence of effort-reward imbalance among nurses: a systematic review and meta-analysis

**DOI:** 10.3389/fpsyg.2024.1425445

**Published:** 2024-07-04

**Authors:** Yujie Zhang, Shanyan Lei, Fang Yang

**Affiliations:** ^1^School of Humanities and Management, Zhejiang Chinese Medical University, Hangzhou, China; ^2^The First Affiliated Hospital, Zhejiang University School of Medicine, Hangzhou, China

**Keywords:** effort-reward imbalance, nurses, systematic review, meta-analysis, incidence

## Abstract

**Introduction:**

To systematically evaluate the incidence of effort-reward imbalance among nurses.

**Method:**

PubMed, Web of Science, Embase, CNKI, WanFang Data, and VIP databases were searched to collect studies on the incidence of effort-reward imbalance among nurses. The search timeframe was from database construction to December 2023. Two researchers independently screened the literature, extracted the data, and evaluated the risk of bias in the included studies. The meta-analysis was performed using Stata 17.1 software.

**Results:**

A total of 60 studies, including 79,644 participants, were included. The prevalence of effort-reward imbalance among nurses was 52.3% (95% *CI*: 44.9–59.7%). In terms of time, the incidence of effort-reward imbalance among nurses before 2010 (26.6, 95%*CI*: 6.8–46.4%) and in 2010–2015 (42.4, 95%*CI*: 32.1–52.8%), 2016–2020 (60.2, 95%*CI*: 49.6–70.7%), and 2021–2023 (65.0, 95%*CI*: 51.5–78.4%) continued to increase. Geographically, Asia (57.4, 95%*CI*: 51.8–63.1%) nurses had a relatively higher prevalence of effort-reward imbalance. In terms of department, the incidence of effort-reward imbalance among nurses was relatively higher in operating rooms (71.8, 95%*CI*: 64.5–79.0%), ICU (64.6, 95%*CI*: 27.7–100.0%), emergency (68.7, 95%*CI*: 62.9–74.5%), and pediatrics (65.8, 95%*CI*: 32.2–99.3%).

**Discussion:**

The prevalence of nurse effort-reward imbalance is high, and there are differences in its prevalence across time, geography, department. Hospital administrators should actively take measures to effectively prevent and reduce the effort-reward imbalance for nurses, especially for nurses in Asia, operating rooms, emergency pediatrics and ICU departments.

**Systematic review registration:**

PROSPERO (CRD42023452428).

## 1 Introduction

In recent years, job stress has received increasing attention. Effort-reward imbalance (ERI) refers to a state of work characterized by high effort and low reward (Colindres et al., 2018). This concept is commonly used to assess job stress. The ERI concept was first introduced by Siegrist (1996). It is based on the idea of the exchange of mutual benefits, whereby an individual gives his or her labor and desires to be rewarded for it (Siegrist et al., 1986). The early ERI model, based on social exchange theory (Cropanzano and Mitchell, 2005), considered ERI a work stressor and explored its impact on stressful consequences and health-related variables. The theory has been adapted twice to form a more complete theoretical system. The research fields wherein it is explored have gradually expanded from industry and commerce, as well as medical and health care, to education, family, sports, and other fields. The empirical research was initially based on model validation, and subsequent studies gradually introduced new mediating and moderating variables, and the theoretical model hypotheses were steadily enriched. This theory has been applied in the field of nursing since 2000 to assess or predict nurses’ psychological well-being, work ability, and other aspects (Bakker et al., 2000).

According to Siegrist’s ERI model, the time and effort that an individual puts into a job is compensated for with money, respect, and opportunities for career advancement. Individuals experience stress when they do not receive what they deserve in return for what they give. Long-term ERI can have several adverse effects on an individual’s physical health, mental health, and work (Siegrist and Li, 2016). The ERI model was originally used to predict the occurrence of cardiovascular disease (Kuper et al., 2002). Studies have shown that chronic ERI can increase one’s risk of cardiovascular disease (Xu et al., 2009) and diabetes (Nordentoft et al., 2020). Mayerl et al. (2021) also suggested that ERI is correlated with depressive symptoms. In addition, chronic excessive ERI can reduce work motivation and satisfaction (Cho et al., 2021) and organizational commitment (Araújo et al., 2022), increase the level of burnout (Nuebling et al., 2013), and lead to a series of behavioral problems such as high absenteeism (Yu et al., 2015) and turnover rates (Leineweber et al., 2021; Fei et al., 2023).

Relevant studies have shown that nurses who believe that their efforts are not recognized and acknowledged can experience psychological imbalances (Satoh et al., 2017). This can lead to gastrointestinal, cardiovascular, and other disorders (Weyers et al., 2006). ERI is also strongly associated with nurses’ mental health (Wu et al., 2023). In addition, nurses’ ERI is associated with quality of work life (Liang et al., 2023), attendance (Trybou et al., 2014), burnout (Li et al., 2021), and turnover intention (Gräske et al., 2023). Nursing staff experience heavy daily workloads, frequent night shifts, and sensitive interpersonal relationships with patients (Xie et al., 2021). This can easily lead to ERI. Therefore, the incidence of ERI among nurses needs more attention.

Although scholars have considered the incidence of ERI and related factors among nurses, studies have produced different results due to the differences in sampling methods, survey areas, and types of nurses among them (specific studies are shown in the Supplementary material). This has resulted in a lack of systematic elucidation of the current situation of ERI among nurses in the existing literature. Therefore, this study was conducted to systematically evaluate the incidence of ERI among nurses and offer theoretical support and practical guidance for effectively preventing or reducing the occurrence of nurses’ ERI.

## 2 Methods

### 2.1 Protocol

The literature search was conducted following the Preferred Reporting Items for Systematic Reviews and Meta-Analyses (PRISMA) guidelines (Moher et al., 2015), and the research protocol was registered in PROSPERO (CRD42023452428).

### 2.2 Search strategy

PubMed, Web of Science, Embase, CNKI, WanFang Data, and VIP databases were searched to collect observational studies on the incidence of ERI among nurses. The timeframe for the search was from the time of database construction to December 31, 2023. The search strategy was (“nurses” OR “nurse clinicians” OR “nurse midwives” OR “nursing staff” OR “health care worker” OR “health worker” OR “health professional” OR “health care professional” OR “health care provider”) AND (“effort-reward imbalance” OR “ERI” OR “effort-reward ratio” OR “ERR” OR “occupational stress” OR “occupational strain” OR “professional tensions”). References of included studies were retrospectively retrieved for additional access to relevant literature.

### 2.3 Study selection

Literature that met the following criteria were included in the study: (1) The study was observational. (2) The study population comprised nurses. (3) The outcome indicator was the incidence of ERI. (4) The assessment tool was the ERI scale. Studies that met the following criteria were excluded: (1) Literature not in English or Chinese. (2) Incomplete data that could not be directly accessed or transformed to obtain the incidence of ERI. (3) Repeated publications or literature with data from the same study. (4) Studies with low quality scores.

### 2.4 Study screening and data extraction

The literature was screened, and data were extracted and cross-checked independently by two researchers (Goldsmith et al., 2007). Any disagreements were resolved through discussion or consultation with a third party. The title and abstract were read for initial screening, and the full text was read and secondarily screened based on the inclusion and exclusion criteria. If needed, the authors of the original studies were contacted by mail and phone to obtain information that was not identified, but was important for this study.

The extracted information included the first author, survey time, survey area, sampling method, age, sex, sample size, number of nurses experiencing ERI, and the incidence rate of ERI. The existence of ERI was determined by the effort-reward imbalance ratio (ERR), which was measured using the subscales of the ERI scale (Siegrist, 1996; Li et al., 2006). ERR = (effort score/reward score) × (11/6), where 11/6 is a correction factor used to correct discrepancies caused by inconsistencies in the number of entries on the effort and reward scales. An ERR >1.0 indicates the presence of ERI. When ERR >1.0, larger ERR values indicate that the nurse is in a more severe state of high effort and low reward (Siegrist et al., 2004).

### 2.5 Quality assessment

Literature quality was evaluated using Cross-sectional Study Evaluation Criteria developed by the Joanna Briggs Institute (2017). The JBI criteria consist of 10 entries that are scored according to the degree of compliance: 0 for non-compliance, 1 for mentioning but not describing in detail, and 2 for a detailed and comprehensive description(Li, 2014; Joanna Briggs Institute, 2017). Generally, a score greater than 70% of the total score is considered high quality (Li, 2014; Joanna Briggs Institute, 2017). The literature quality evaluation was performed independently by two researchers back-to-back (Zhang Q. et al., 2024). For controversial literature, a third party was consulted to assist in judgment and reaching a consensus (Zhang Q. et al., 2024).

### 2.6 Statistical analysis

Stata 17.0 software (Stata Corporation, College Station, TX, United States) was used for statistical analysis. The incidence of effort-reward imbalance was used as the statistical effect size and a 95% CI was provided. The *χ^2^* test was used to analyze the heterogeneity between the outcomes of the included studies (test level of α = 0.1), while *I^2^* was used as an indicator to test the heterogeneity of the included studies. If there was no statistical heterogeneity among the study results (*I^2^* < 50%), meta-analysis was performed using a fixed-effects model. If there was statistical heterogeneity among the study results (*I^2^* > 50%), meta-analysis was performed using a random-effects model. The source of heterogeneity was analyzed using an article-by-article exclusion of literature method. Funnel plots, Begg’s test, and Egger’s test were used to determine the presence of publication bias. Subgroup analyses were conducted by population different gender, age, education, title, years of service, marital status, publication time, and continent to further explore possible influencing factors. The level of significance was alpha = 0.05.

## 3 Results

### 3.1 Selection of studies and basic characteristics

**Figure 1:**
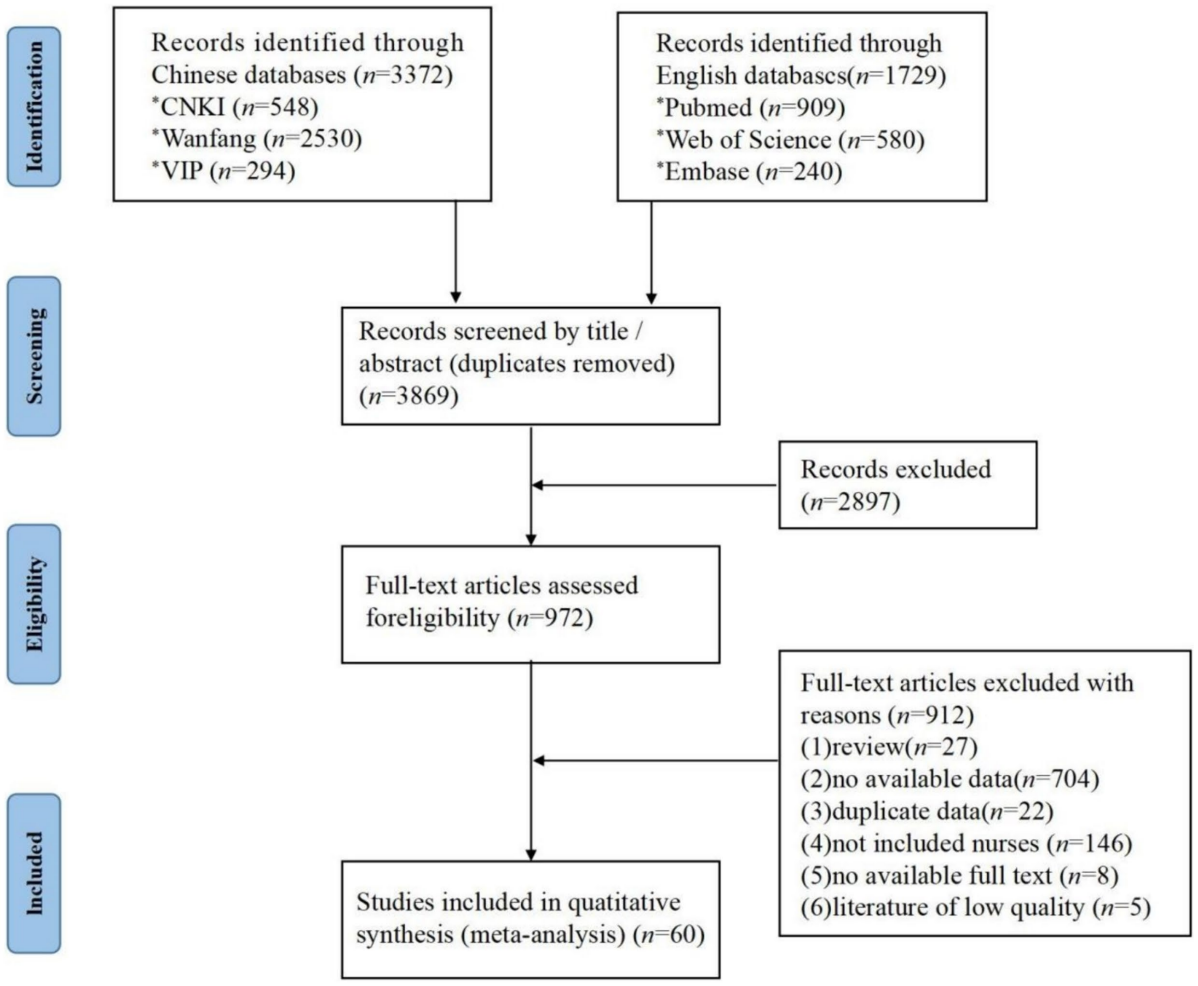
Flow chart of literature screening.

In total, 5,101 relevant articles were obtained from the search. After initial screening and full-text reading, 60 studies were selected. As presented in Figure 1.

The timeframe of the included studies ranged from 2002 to 2021. The sampling method was based on convenience sampling in 35 studies. Regarding study setting, 4 articles concerned emergency department nurses, 3 articles concerned operating room nurses, 3 articles concerned pediatric nurses, and 47 documents were not classified. The effective sample size totaled 79,644 cases and the number of ERI occurrences was 31,978. The incidence of ERI ranged from 0.81 to 97.37%. The basic characteristics of included studies are reported in Table 1.

**Table 1:** Characteristics of the studies included in the meta-analysis.

Authors (Publication year)	Survey time	Country	Sampling method	Age (years) (*Mean ± SD*)	Sample size *(n)*	Male/Female *(n)*	Number of Incidence *(n)*	Incidence rate (%)	JBI score
Hasselhorn (2004)	2002–2003	Europe	C	NR	21,729	NR	2,926	13.47	14
Kluska (2004)	NR	Canada	R	32.85 ± 8.64	112	2/110	27	24.11	16
Lavoie-Tremblay (2008)	2006	Canada	C	22.6 ± 1.28	309	11/298	181	58.58	16
Spence Laschinger (2008)	NR	Canada	R	48.04 ± 10.21	134	5/129	14	10.45	16
Wang (2010)	NR	China	C	29.81 ± 6.20	470	NR	150	31.91	14
Aparecida (2010)	2004–2005	Brazil	C	NR	696	NR	54	7.76	14
Xie (2010)	2008.6	China	CS,R,P	32.11	527	NR	137	26.00	15
Herin (2011)	2006	France	R,S	NR	2,176	NR	753	34.6	19
Chen (2012)	2011.3–5	China	R,S,P	31.40 ± 8.10	384	NR	142	36.98	14
Enberg Birgit (2012)	2002	Sweden	C	NR	198	26/172	42	21.21	17
Gao (2012)	2009.4–6	China	CS,R	35.01 ± 9.33	1,437	NR	378	26.30	14
Fischer (2012)	2009	Brazil	CS	35.5 ± 8.1	494	NR	4	0.81	17
Liu (2012)	NR	China	C	24.91 ± 2.91	338	14/324	270	79.88	15
Huang (2013)	NR	China	R,S	NR	980	NR	340	34.69	19
Lee (2013)	2006	American	C	46.7 ± 8.7	304	278/92	96	31.58	17
Liu (2013)	NR	China	R,P	29.08 ± 6.68	1,145	NR	656	57.29	14
Zhu (2013)	NR	China	C	30.48 ± 7.59	275	3/272	161	58.55	15
Li (2013)	NR	Germany, Italy, et al.	C	36.66 ± 8.64	7,990	NR	2,340	29.29	15
Fang (2014)	2012.8	China	C	38.86 ± 6.29	233	0/ 233	89	38.20	16
Nourry (2014)	2007–2008	France	C	46.2 ± 7.4	296	32/264	52	17.57	14
Liu (2014)	NR	China	S	NR	589	63/526	411	69.78	14
Yokoyama (2014)	NR	Japan	R	38.2 ± 12.3	342	45/297	291	85.09	19
Yuan (2015)	NR	China	R,S	NR	397	19/378	253	63.73	15
Lin (2015)	2005.7–10	China	C	NR	654	0/654	214	32.72	15
Julia Claire (2015)	2014.4–9	American	C	45.9 ± 11.9	238	NR	182	76.47	17
Shi (2015)	NR	China	CS,S	NR	488	20/468	359	73.57	16
He (2016)	2015.2–7	China	C	31.29 ± 6.25	810	9/801	559	69.01	17
Lu (2016)	NR	China	CS	NR	102	0/102	69	67.65	15
Martinez (2017)	2009–2011	Brazil	C	35.9 ± 9.0	304	61/243	296	97.37	16
Chen (2017)	2016.1–2	China	S,P	NR	715	NR	290	40.56	14
Wang (2017)	2015.9–2016.4	China	C	27.69 ± 5.74	382	163/366	161	42.15	15
Du (2017)	2016.3–9	China	C	NR	424	13/411	218	51.42	16
de Oliveira (2017)	2010.4–2011.12	Brazil	C	NR	675	NR	382	56.59	16
Liu (2017)	NR	China	C	31.82 ± 9.22	704	8/696	471	66.90	15
Wang (2017)	2016.4–6	China	CS	NR	679	0/679	357	52.58	18
Pinhatti (2018)	2016.11–2017.1	Brazil	S	NR	285	71/214	227	79.65	17
Li (2018)	2017.3–6	China	CS,R	37.10 ± 6.10	243	11/131	175	72.02	16
Zaree (2018)	2015.9–2016.6	Iran	C	31.7 ± 6.4	379	42/337	312	82.32	17
Liang (2018)	2017.12–2018.2	China	C	33.26 ± 11.32	671	34/637	423	63.04	16
Colindres (2018)	2014.10–2015.2	Ecuadorian	C	35.4	333	39/290	68	20.42	18
Lua (2018)	2011–2012	Brazil	R	NR	436	NR	296	67.89	18
Deng (2018)	2017.7–8	China	C	NR	396	9/387	160	40.40	14
Salem (2018)	2016.2–7	Egypt	CS	NR	204	12/192	148	72.55	17
Fang (2019)	NR	China	R,S	32.84 ± 7.45	236	6/230	181	76.69	18
Chai (2020)	2019.9–12	China	C	NR	125	15/100	91	72.80	16
Kong (2020)	2019	China	C	34.06 ± 8.02	1,077	23/1054	285	26.46	16
Bardhan (2019)	2015.9–2017.2	American	C	33.04 ± 9.79	42	13/29	39	92.86	14
Yasira (2020)	2019.1–3	China	C	38.80 ± 7.10	487	42/445	126	25.87	15
Wang (2020)	2019.4–6	China	C	32.00 ± 8.75	206	18/188	98	47.57	16
Babazadeh (2021)	2020.8–9	Iran	R,S	33.14 ± 8.45	203	76/127	164	80.79	19
Gao (2021)	2020.11–2021.1	China	CS	NR	679	NR	452	66.57	16
Su (2021)	2019.3–2020.6	China	CS	24.57 ± 1.80	161	19/142	105	65.22	16
Tzenetidis (2021)	2020.4–5	Greece	C	35.68 ± 7.07	200	128/72	151	75.50	15
Tian (2021)	2018.7–8	China	C	NR	17,582	1803/15779	10,489	59.66	14
Li (2021)	2019	China	CS,R,S	35.04 ± 8.45	756	163/588	559	73.94	18
Martinez (2022)	2018.10–2019.3	Brazil	C	NR	1,238	195/1043	143	11.55	16
Gao (2022)	2020.10–2021.5	China	C	NR	708	0/708	366	51.69	18
Yan (2022)	2021.1–7	China	C	32.56 ± 6.57	1,536	50/1486	1,355	88.22	18
Gustavsson (2022)	NR	Poland	C	34.48 ± 10.40	117	NR	97	82.91	15
An (2023)	2018.7–8	China	C	NR	3,614	3377/237	2,143	59.30	17

NR, Not Reported; C, Convenient sampling; CS, Cluster sampling; R, Random sampling; S, Stratified sampling; SS, Systemic sampling; P, Probability sampling.

### 3.2 Quality assessment

The quality scores of the included studies ranged from 14 to 19, with a mean score of 15.98. None of the scores were less than 70% of the total score, and most of them were medium- to high-quality studies. However, the scores for the selection of sampling methods and the measures to verify the authenticity of information were low, with the average scores of the entries being only 0.72 and 1.08. The results of quality assessment are reported in Supplementary materials 1.

### 3.3 Incidence of effort-reward imbalance in nurses

**Figure 2:**
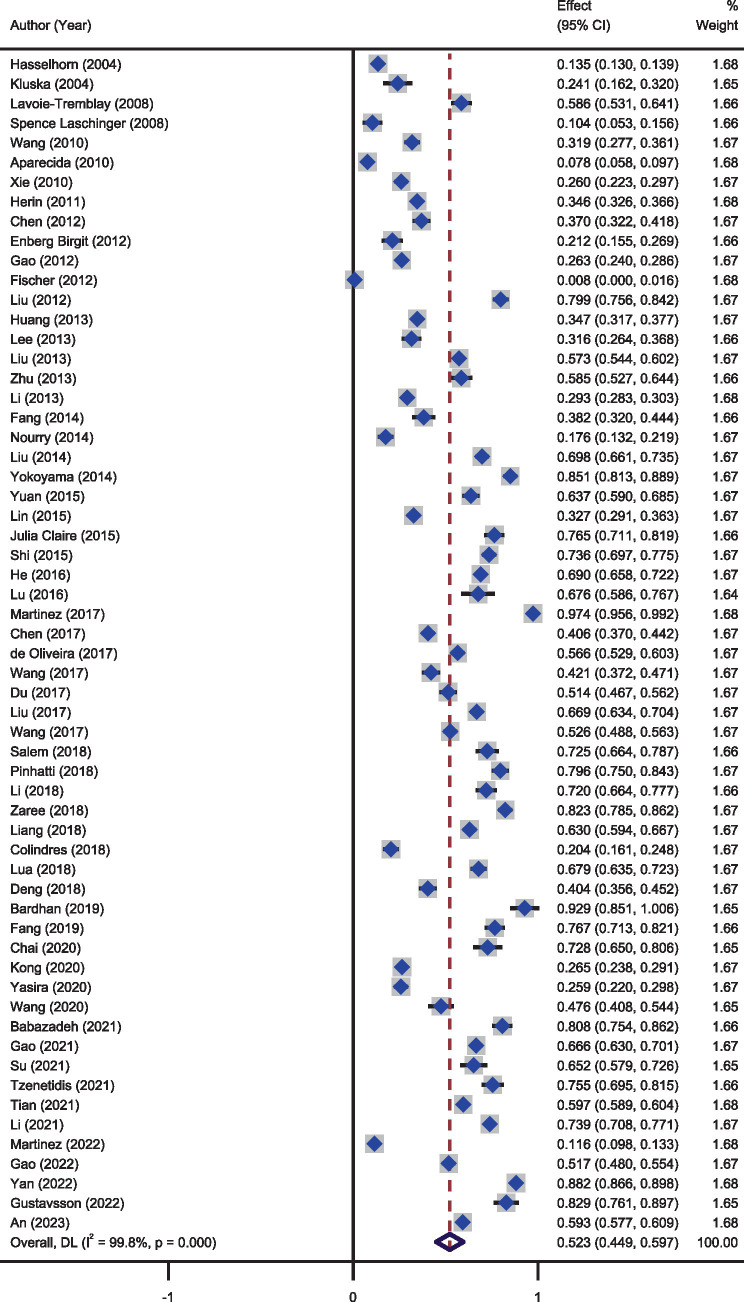
A forest graph showing the pooled estimates of the incidence of effort-reward imbalance.

The results of the heterogeneity test showed large heterogeneity among the results of the different studies (*I^2^* = 99.8%, *p* < 0.01). Meta-analysis of the random effects model showed that the incidence of effort-reward imbalance among nurses was 52.3% (95%*CI*: 44.9–59.7%). As presented in Figure 2.

### 3.4 Publication bias and sensitivity analysis

**Figure 3:**
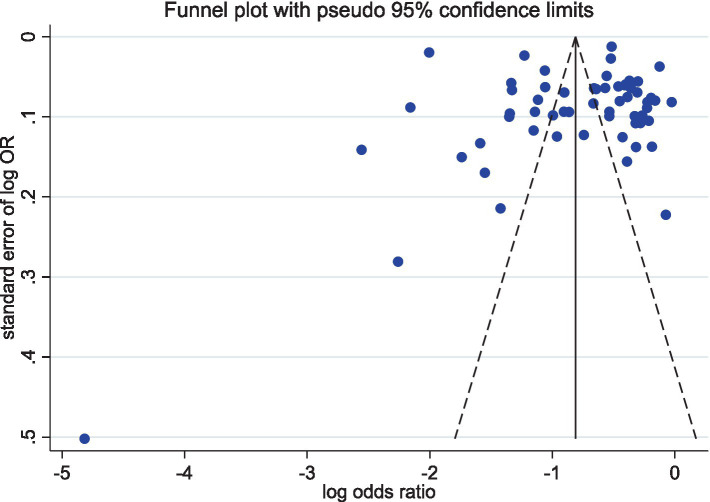
Funnel plot.

As shown in Figure 3, there was symmetry in the distribution on both sides of the funnel plot. The *p*-values for Egger’s test was 0.570 (*p* > 0.05), respectively. These methods suggest that publication bias is unlikely.

**Figure 4:**
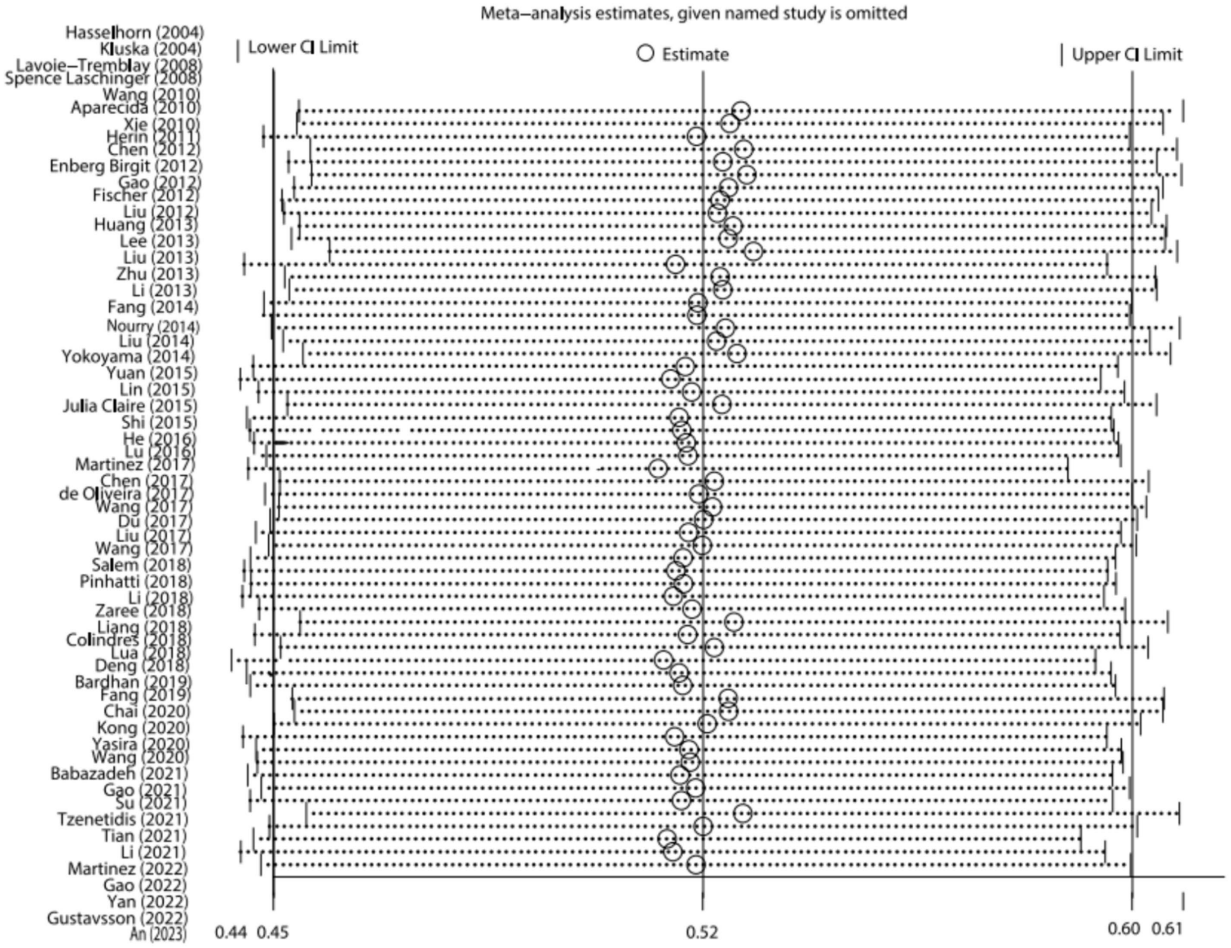
Sensitivity analysis.

Sensitivity analysis of the included studies was performed using a stepwise exclusion method. Sequential exclusion of the studies revealed that the total combined value was at most 0.9 percentage points higher than before exclusion, and the results were not significantly different from the total combined estimate. As presented in Figure 4. This indicates that the sensitivity of this study was low and the results were more stable.

### 3.5 Subgroup analysis

Subgroup analyses showed that, grouped by publication year, the incidences of ERI among nurses before 2010 and in 2010–2015, 2016–2020, and 2021–2023 were 26.6, 42.4, 60.2, and 65.0%, respectively (*p* < 0.05). The incidence of nurse ERI was 57.4, 38.8, 49.0, and 42.7% in Asia, Europe, North America, and South America, respectively (*p* < 0.05). The incidence of ERI among Chinese nurses in emergency medicine, operating rooms, pediatrics, ICU, and no classification was 68.7, 71.8, 65.8, 64.6, and 49.0%, respectively (*p* < 0.05). There was no significant correlation between the incidence of ERI among nurses and gender, age, education, years of service, sampling method, or marital status (*p* > 0.05). The results of subgroup analysis are reported in Table 2.

**Table 2:** Incidence rate of effort-reward imbalance by demographic characteristics.

Subgroups	Categories	No. of studies	Sample size	Heterogeneity		Incidence rate (%)	95%*CI*	*Q*	*p*
				*I^2^* (%)	*p*				
Gender	Male	4	2030	55.2	0.08	65.4	(59.0, 71.7)	0.31	0.58
	Female	4	16,369	97.3	<0.001	69.4	(56.5, 82.3)		
Age(year)	≤25	3	3,802	95.1	<0.001	61.1	(45.4, 76.9)	2.24	0.33
	26–35	3	11,264	48.5	0.14	62.4	(59.5, 65.2)		
	>35	5	3,871	84.4	<0.001	69.3	(60.6, 77.9)		
Educational level	Junior college and below	7	11,158	96.8	<0.001	59.5	(50.1, 68.9)	<0.01	0.97
	Undergraduate or above	7	9,168	92.9	<0.001	59.2	(52.2, 66.3)		
Years of service	≤5	5	10,847	85.2	<0.001	62.6	(55.4, 69.9)	5.23	0.07
	6–10	4	4,861	70.6	0.02	68.3	(61.9, 74.4)		
	>10	5	2,974	92.1	<0.001	77.8	(66.9, 88.6)		
Marital status	Unmarried/other	5	11,933	98.3	<0.001	64.7	(51.3, 78.1)	0.56	0.46
	Married	5	7,181	70.0	0.01	59.1	(53.3, 64.9)		
Publication year	<2010	4	22,284	98.9	<0.001	26.6	(6.8, 46.4)	15.45	0.001
	2010–2015	22	20,651	99.7	<0.001	42.4	(32.1, 52.8)		
	2016–2020	23	9,915	99.4	<0.001	60.2	(49.6, 70.7)		
	2021–2023	11	26,794	99.8	<0.001	65.0	(51.5, 78.4)		
Continent	Asia	39	41,622	99.3	<0.001	57.4	(51.8, 63.1)	9.54	0.02
	Europe	7	32,706	99.7	<0.001	38.8	(28.0, 49.6)		
	North America	6	1,139	99.0	<0.001	49.0	(24.2, 73.7)		
	South America	8	4,461	99.9	<0.001	42.7	(13.8, 71.6)		
Sampling method	Convenient sampling	35	65,439	99.9	<0.001	51.9	(42.0, 61.9)	0.01	0.91
	other	25	14,205	99.8	<0.001	52.9	(39.9, 65.9)		
Type of nurses	Department of emergency	4	21,363	96.3	<0.001	68.7	(62.9, 74.5)	21.99	<0.001
	Operation room	3	464	63.8	0.06	71.8	(64.5, 79.0)		
	Department of pediatrics	3	459	99.2	<0.001	65.8	(32.2, 99.3)		
	ICU	3	715	98.7	<0.001	64.6	(27.7, 100.0)		
	No categories	47	53,930	99.8	<0.001	49.0	(41.4, 56.6)		

## 4 Discussion

The results of this study showed that the prevalence of ERI among nurses ranged from 0.81 to 97.37%. The results varied considerably between studies, which may be closely related to differences in sample size, sampling methods, and types of nurses. It is worth noting that, by quantitatively combining the data from 60 studies, the findings of the meta-analysis showed that the prevalence of ERI among nurses was 52.3%. This indicates that more than half of nurses are in a state of ERI.

The State of the World’s Nursing 2020 report published by the World Health Organization shows that there are 27.9 million nursing practitioners globally, of which 19.3 million are professional nurses (World Health Organization, 2020). The total stock increased by 4.7 million between 2013 and 2018. The number of nurses per 1,000 people in the Americas amounted to 8.34, compared with 7.93 in the European region in 2018 (World Health Organization, 2020). By the end of 2022, the total number of registered nurses in China exceeded 5.2 million, with approximately 3.7 registered nurses per 1,000 people (The State Council the People's Republic of China, 2023). While the total number of nurses has increased in recent years, they are nevertheless in short supply, and their number still varies greatly from country to country and from region to region.

In addition to this, on the one hand, residents’ income structure has gradually become more diversified and optimized over time, and their consumption structure has been transforming and upgrading. Thus, their demand for diversified nursing services is increasing. Given the shortage of nurses and increasing demand for nursing care, the work pressure on nurses will continue to increase. On the other hand, nursing is an innately stressful and high-risk profession (Zhang Y. et al., 2024), characterized by stressful conditions such as confronting suffering and death (Kostka et al., 2021), heavy workloads (Norful et al., 2022), workplace violence (Liu et al., 2022), forced overtime hours (Geiger-Brown and Lipscomb, 2010), and more. Compared to other professionals, nurses inherently face a higher level of stress and are required to put in more effort (Shechter et al., 2020). Although nurses receive appropriate financial remuneration, welfare benefits, and respect for their work, most believe that there is an imbalance between their own substantial efforts and the accompanying reward received.

More notably, the results of the subgroup analysis showed that the incidence of ERI among nurses has gradually increased over time, reaching 65.0% between 2021 and 2023. This suggests that nurses’ ERI needs to be alleviated urgently. This increasing ERI may be related to the prevalence of COVID-19 since 2020. During the fight against the COVID-19 pandemic, nurses were responsible for collecting specimens, monitoring vital signs, providing life care, and many other aspects of nursing work (Zhang et al., 2021; Al-Amer et al., 2022). This required more effort from nurses than ever before, and their work was accompanied by increased pressures from multiple sources, including increased workload, fear of bringing the virus home, and possible infection (Walton et al., 2020; Wang et al., 2022).

Regionally, the prevalence of ERI is higher among nurses in Asia. This is broadly in line with the results of the State of the World’s Nursing Report 2020. The results of this report show that in 2018, there was a global nursing shortage of 5.9 million nurses, and the nursing shortage was mainly in low- and middle-income countries such as those in Africa, Southeast Asia, and Latin America (World Health Organization, 2020). The Asia areas surveyed in the relevant literature included in this study are mainly in China, totaling 35 articles. In China, the work content of nurses is inherently complicated, and their working hours are irregular. Simultaneously, as healthcare workers, nurses must have a strong sense of service, dedication, and empathy (Tucker et al., 2015). This requires nurses to exert immense effort in terms of managing their workload, working hours, psychology, and emotions (Zhang et al., 2023). Additionally, in China, it is more difficult for nurses to be promoted because of the large number of nurses overall, the limited number of places where jobs are available, and limited opportunities for promotion. In terms of employment type, most nurses in China are contract-based nurses whose remuneration and job stability are poor (Shang et al., 2014). Further, compared to doctors, nurses in China face large gaps in their working environments, salary levels, promotion prospects, and social status (Yun et al., 2010; Zhang et al., 2014). With the implementation of the “equal wages for equal work” policy and the “people-oriented” flexible management model, in recent years, nurses’ remuneration, welfare benefits, participation in decision-making, and work control have improved to a certain extent in China. All of this has, to a certain extent, helped reduce the effort-reward ratio among Chinese nurses. However, overall, the incidence of ERI is relatively high.

Subgroup analyses showed a higher prevalence of ERI among the operating room, emergency, pediatric, and ICU department nurses (71.8, 68.7, 65.8, and 64.6%, respectively). These nurses have heavier daily workloads and higher overall workloads (Zhou and Gong, 2015; Elder et al., 2020). They are usually in a state of high intensity, pressure, and risk for long periods, and often bear more work pressure than nurses in other departments (Ma et al., 2022). Operating room and ICU department nurses work in a more closed environment, pediatric nurses work under high stress due to the specificity of the population they serve, whereas emergency department nurses are constantly exposed to critical, sudden, and variable work environments. However, the reward for nurses in the operating room, emergency, pediatric, and ICU departments is not significantly different from that of nurses in other departments, which increases the incidence of ERI. This is consistent with ERI theory, which posits that the more effort one puts in at work, the more one desires in return (Siegrist, 1996), and that the degree of ERI is exacerbated when the corresponding reward is not received.

Based on the results of our study, to reduce or prevent ERI in nurses, some potentially effective interventions are necessary to implement. According to the ERI model, efforts mainly include workload, time pressure, overtime, and so on. Rewards mainly include pay, respect, job security, and job control. The balance between efforts and rewards can be struck by reducing nurses’ efforts or increasing their rewards. Nursing managers can involve nurses in decisions about organizational change and respect nurses’ right to participate and have a voice in the management process (Kluska et al., 2004). This may increase nurses’ sense of control over their work and make them feel respected and recognized. At the same time, the income of nurses should be increased appropriately and a sound workplace violence response program should be put in place to reduce the occurrence of such violence (Tian et al., 2021). Secondly, more opportunities should be provided for nurses’ career development, such as opportunities to upgrade their academic qualifications and pursue further study, which will help them meet their personal development needs and increase their sense of work-derived rewards.

Moreover, there is a need to replenish the number of nurses in a timely manner and reduce the intensity of nurses’ work by increasing their number. Meanwhile, nursing managers should ensure more reasonable and flexible scheduling, establish clear and more reasonable division of tasks, and reduce the occurrence of delays in leaving work (Yan et al., 2022). This can lead to optimized workflow and reduced time pressure on nurses’ work. Based on the findings of this study, nursing administrators should pay special attention to operating room, emergency, pediatric, and ICU department nurses; they should provide appropriate consideration to these nurses in terms of salary distribution, incentives, and promotion to fully reflect the value of their labor. These interventions could help restore the balance between effort and reward and prevent or reduce ERI for nurses.

## 5 Limitations

First, from the perspective of evidence-based medicine, research evidence on this topic needs to be as comprehensive as possible, and it is certainly more scientific and comprehensive to search relevant literature in all languages. However, considering the limited search scope, it was difficult to access non-Chinese and non-English literature. Therefore, we included only Chinese and English literature in this study. This may limit the generalizability of our results. Second, there was a high degree of heterogeneity in the included studies, owing to the characteristics of using a single-arm meta-analysis. This heterogeneity could not be adequately adjusted through subgroup analysis, potentially due to the fact that the included studies were based on different continents, sampling methods, types of nurses, etc. This is consistent with the notion that it is difficult to avoid heterogeneity in the meta-analysis of epidemiologic surveys (Long et al., 2014; Andersen et al., 2023). When heterogeneity is high, it exceeds random error, which may influence the reliability of the study results.

Third, the literature included in this study consisted of cross-sectional studies. Participants in cross-sectional studies may rely on their memories to report past behaviors or experiences. This raises the possibility of recall bias. Additionally, the self-reporting process may be somewhat subjective, and may therefore influence the accuracy of the results of this study. Fourth, in terms of sampling methodology, most of the included studies were based on convenience sampling, which did not allow for strict control of the study population. Further, although the data were anonymous and confidential, some nurses may have avoided providing accurate information due to lack of trust or forgetfulness. This could affect the accuracy of the results of this study to some extent. In addition, some of the included studies lacked or did not mention measures to verify the authenticity of the information. This may impact the accuracy of the study results.

## 6 Conclusion

The prevalence of ERI is high among nurses and has increased gradually over time. In addition, there are differences in the incidence of ERI among nurses according to continent and nursing department. Specifically, the incidence of ERI is relatively high among nurses in Asia, as well as among operating room, emergency, pediatric, and ICU department nurses. Our study clarifies the current incidence of ERI among nurses and identifies certain nurse groups that need special attention. This study suggests that nursing administrators should be deeply aware of the importance and seriousness of the ERI problem among nurses and that there is a great need to implement scientific interventions. This meta-analysis study examined nurses from different countries, rendering the results more generalizable. Thus, it serves not only as an examination of the results of existing studies, but also as a reference for future nursing management practice, research, and policy.

## Data availability statement

The raw data supporting the conclusions of this article will be made available by the authors, without undue reservation.

## Author contributions

YZ: Conceptualization, Data curation, Investigation, Methodology, Project administration, Software, Validation, Writing – original draft, Writing – review & editing. SL: Data curation, Investigation, Writing – review & editing. FY: Funding acquisition, Project administration, Supervision, Validation, Writing – review & editing.
