# Supplementary material for: Incidence of effort-reward imbalance among nurses: a systematic review and meta-analysis
Source: Front Psychol. 2024 Jul 4;15:1425445. doi: 10.3389/fpsyg.2024.1425445 (PMC11255848; doi:10.3389/fpsyg.2024.1425445)
Supplement: Supplementary file 1 [file Table_1.DOC]

**Supplementary Materials**

Supplementary material 1 Results of bias risk assessment of included studies

| Authors | ① | ② | ③ | ④ | ⑤ | ⑥ | ⑦ | ⑧ | ⑨ | ⑩ | JBI score |
| --- | --- | --- | --- | --- | --- | --- | --- | --- | --- | --- | --- |
| Hasselhorn (2004) | 2 | 0 | 2 | 1 | 2 | 1 | 0 | 2 | 2 | 2 | 14 |
| Kluska (2004) | 2 | 2 | 1 | 2 | 2 | 1 | 0 | 2 | 2 | 2 | 16 |
| Lavoie-Tremblay (2008) | 2 | 0 | 2 | 2 | 0 | 2 | 2 | 2 | 2 | 2 | 16 |
| Spence Laschinger (2008) | 2 | 2 | 1 | 2 | 2 | 1 | 0 | 2 | 2 | 2 | 16 |
| Wang (2010)^20^ | 2 | 2 | 0 | 2 | 1 | 0 | 1 | 2 | 2 | 2 | 14 |
| Aparecida (2010) | 2 | 0 | 2 | 1 | 0 | 1 | 2 | 2 | 2 | 2 | 14 |
| Xie (2010)^33^ | 2 | 1 | 0 | 2 | 2 | 0 | 2 | 2 | 2 | 2 | 15 |
| Herin (2011) | 2 | 2 | 2 | 1 | 2 | 2 | 2 | 2 | 2 | 2 | 19 |
| Chen (2012)^19^ | 2 | 2 | 1 | 2 | 1 | 1 | 0 | 1 | 2 | 2 | 14 |
| Enberg Birgit (2012) | 2 | 1 | 1 | 1 | 2 | 2 | 2 | 2 | 2 | 2 | 17 |
| Gao (2012)^27^ | 2 | 2 | 0 | 2 | 0 | 0 | 2 | 2 | 2 | 2 | 14 |
| Fischer (2012) | 2 | 1 | 1 | 1 | 2 | 2 | 2 | 2 | 2 | 2 | 17 |
| Liu (2012)^11^ | 2 | 0 | 1 | 2 | 2 | 1 | 1 | 2 | 2 | 2 | 15 |
| Huang (2013)^17^ | 2 | 2 | 2 | 2 | 2 | 2 | 1 | 2 | 2 | 2 | 19 |
| Lee (2013) | 2 | 0 | 1 | 2 | 2 | 2 | 2 | 2 | 2 | 2 | 17 |
| Liu (2013)^18^ | 2 | 2 | 0 | 2 | 0 | 2 | 0 | 2 | 2 | 2 | 14 |
| Zhu (2013)^16^ | 2 | 0 | 2 | 2 | 2 | 0 | 1 | 2 | 2 | 2 | 15 |
| Li (2013) | 2 | 0 | 1 | 2 | 1 | 1 | 2 | 2 | 2 | 2 | 15 |
| Fang (2014)^31^ | 2 | 0 | 2 | 2 | 2 | 0 | 2 | 2 | 2 | 2 | 16 |
| Nourry (2014) | 2 | 0 | 2 | 2 | 1 | 1 | 0 | 2 | 2 | 2 | 14 |
| Liu (2014)^15^ | 2 | 1 | 1 | 2 | 2 | 0 | 1 | 1 | 2 | 2 | 14 |
| Yokoyama (2014) | 2 | 2 | 2 | 2 | 2 | 1 | 2 | 2 | 2 | 2 | 19 |
| Yuan (2015)^14^ | 2 | 2 | 1 | 2 | 1 | 0 | 1 | 2 | 2 | 2 | 15 |
| Lin (2015)^35^ | 2 | 0 | 1 | 2 | 2 | 0 | 2 | 2 | 2 | 2 | 15 |
| Julia Claire (2015) | 2 | 0 | 2 | 2 | 2 | 1 | 2 | 2 | 2 | 2 | 17 |
| Shi (2015)^22^ | 2 | 1 | 1 | 2 | 1 | 2 | 1 | 2 | 2 | 2 | 16 |
| He (2016)^13^ | 2 | 0 | 2 | 2 | 2 | 2 | 1 | 2 | 2 | 2 | 17 |
| Lu (2016)^25^ | 2 | 1 | 1 | 2 | 1 | 2 | 0 | 2 | 2 | 2 | 15 |
| Martinez (2017) | 2 | 0 | 1 | 2 | 2 | 1 | 2 | 2 | 2 | 2 | 16 |
| Chen (2017)^8^ | 2 | 1 | 2 | 1 | 0 | 0 | 2 | 2 | 2 | 2 | 14 |
| Wang (2017)^10^ | 2 | 0 | 2 | 2 | 1 | 2 | 1 | 1 | 2 | 2 | 15 |
| Du (2017)^24^ | 2 | 0 | 2 | 2 | 1 | 2 | 1 | 2 | 2 | 2 | 16 |
| de Oliveira (2017) | 2 | 0 | 1 | 1 | 2 | 2 | 2 | 2 | 2 | 2 | 16 |
| Liu (2017)^12^ | 2 | 0 | 2 | 2 | 2 | 0 | 1 | 2 | 2 | 2 | 15 |
| Wang (2017)^9^ | 2 | 2 | 2 | 2 | 1 | 2 | 1 | 2 | 2 | 2 | 18 |
| Pinhatti (2018) | 2 | 1 | 2 | 2 | 1 | 1 | 2 | 2 | 2 | 2 | 17 |
| Li (2018)^6^ | 2 | 2 | 2 | 2 | 1 | 0 | 1 | 2 | 2 | 2 | 16 |
| Zaree (2018) | 2 | 0 | 2 | 2 | 2 | 1 | 2 | 2 | 2 | 2 | 17 |
| Liang (2018)^23^ | 2 | 2 | 2 | 2 | 1 | 0 | 1 | 2 | 2 | 2 | 16 |
| Colindres (2018) | 2 | 0 | 2 | 2 | 2 | 2 | 2 | 2 | 2 | 2 | 18 |
| Lua (2018) | 2 | 2 | 2 | 2 | 1 | 1 | 2 | 2 | 2 | 2 | 18 |
| Deng (2018)^7^ | 2 | 0 | 2 | 1 | 2 | 0 | 1 | 2 | 2 | 2 | 14 |
| Salem (2018) | 2 | 1 | 2 | 2 | 2 | 1 | 2 | 1 | 2 | 2 | 17 |
| Fang (2019)^34^ | 2 | 2 | 2 | 2 | 2 | 0 | 2 | 2 | 2 | 2 | 18 |
| Chai (2020)^4^ | 2 | 0 | 2 | 1 | 2 | 2 | 1 | 2 | 2 | 2 | 16 |
| Kong (2020)^30^ | 2 | 0 | 1 | 2 | 2 | 1 | 2 | 2 | 2 | 2 | 16 |
| Bardhan (2019) | 2 | 0 | 2 | 2 | 0 | 0 | 2 | 2 | 2 | 2 | 14 |
| Yasira (2020)^29^ | 2 | 0 | 2 | 2 | 1 | 0 | 2 | 2 | 2 | 2 | 15 |
| Wang (2020)^5^ | 2 | 0 | 2 | 2 | 2 | 1 | 1 | 2 | 2 | 2 | 16 |
| Babazadeh (2021) | 2 | 2 | 2 | 2 | 2 | 1 | 2 | 2 | 2 | 2 | 19 |
| Gao (2021)^26^ | 2 | 0 | 2 | 2 | 2 | 2 | 0 | 2 | 2 | 2 | 16 |
| Su (2021)^21^ | 2 | 0 | 2 | 2 | 1 | 1 | 2 | 2 | 2 | 2 | 16 |
| Tzenetidis (2021) | 2 | 0 | 2 | 2 | 0 | 1 | 2 | 2 | 2 | 2 | 15 |
| Tian (2021)^28^ | 2 | 0 | 0 | 2 | 1 | 1 | 2 | 2 | 2 | 2 | 14 |
| Li (2021)^3^ | 2 | 1 | 2 | 2 | 2 | 1 | 2 | 2 | 2 | 2 | 18 |
| Martinez (2022) | 2 | 0 | 1 | 2 | 2 | 1 | 2 | 2 | 2 | 2 | 16 |
| Gao (2022)^2^ | 2 | 0 | 2 | 2 | 2 | 2 | 2 | 2 | 2 | 2 | 18 |
| Yan (2022)^32^ | 2 | 0 | 2 | 2 | 2 | 2 | 2 | 2 | 2 | 2 | 18 |
| Gustavsson (2022) | 2 | 0 | 2 | 1 | 1 | 1 | 2 | 2 | 2 | 2 | 15 |
| An (2023)^1^ | 2 | 0 | 1 | 2 | 2 | 2 | 2 | 2 | 2 | 2 | 17 |

**Additional References**

1. Hasselhorn HM, Tackenberg P, Peter R. Effort-reward imbalance among nurses in stable countries and in countries in transition. *Int J Occup Environ Health*. (2004) 10:401-408. doi: 10.1179/oeh.2004.10.4.401.
2. Kluska KM, Laschinger HK, Kerr MS. Staff nurse empowerment and effort-reward imbalance. *Nurs Leadersh (Tor Ont)*. (2004) 17:112-128. doi: 10.12927/cjnl.2004.16247.
3. Lavoie-Tremblay M, O'Brien-Pallas L, Gélinas C, Desforges N, Marchionni C. Addressing the turnover issue among new nurses from a generational viewpoint.*J Nurs Manag*. (2008) 16:724-733. doi: 10.1111/j.1365-2934.2007.00828.x.
4. Spence Laschinger HK, Finegan J. Situational and dispositional predictors of nurse manager burnout: a time-lagged analysis. *J Nurs Manag*. (2008) 16:601-607. doi: 10.1111/j.1365-2834.2008.00904.x.
5. Wang X, Zhou P, Ren W, Li L. Influence of effort-reward imbalance on turnover intention in nurses. *J Shanghai Jiaotong University*. (2010) 30:459-62.
6. Silva AA, Souza JM, Borges FN, Fischer FM. Health-related quality of life and working conditions among nursing providers. *Rev Saude Publica*. (2010) 44:718-725. doi: 10.1590/s0034-89102010000400016.
7. Xie Z, Wang A, Chen B. Nurse burnout and its association with occupational stress in a cross-sectional study in Shanghai. *J Adv Nurs*. (2011) 67:1537-46. doi: 10.1111/j.1365-2648.2010.05576.x.
8. Herin F, Paris C, Levant A, Vignaud MC, Sobaszek A, Soulat JM. Links between nurses' organisational work environment and upper limb musculoskeletal symptoms: independently of effort-reward imbalance! The ORSOSA study. *Pain*. (2011) 152:2006-2015. doi: 10.1016/j.pain.2011.04.018.
9. Chen C, Hua Y, Nie F, Wang X, Zhong B. Relationship between effort-reward imbalance and depression among nurses in Wuhan. *J Nurs Sci*. (2012) 27:77-8. doi: 10.3870/hlxzz.2012.17.077.
10. Birgit E, Gunnevi S, Ann Ö. Work experiences among nurses and physicians in the beginning of their professional careers - analyses using the effort-reward imbalance model. *Scand J Caring Sci*. (2013) 27:36-43. doi: 10.1111/j.1471-6712.2012.00997.x.
11. Gao Y, Pan B, Sun W, Wu H, Wang J, Wang L. Anxiety symptoms among Chinese nurses and the associated factors: a cross sectional study. *BMC Psychiatry*. (2012) 12:141. doi: 10.1186/1471-244X-12-141.
12. Fischer FM, Martinez MC. Individual features, working conditions and work injuries are associated with work ability among nursing professionals. *Work*. (2013) 45:509-517. doi: 10.3233/WOR-131637.
13. Liu J, Liu H. Research on the correlation between effort-reward imbalance and turnover intention among contract nurses. *J Nurs Admin*. (2012) 12:7-9.
14. Huang L. Association study of job stress, burnout and presenteeism in health care workers. *Fudan university*. (2013). MA thesis.
15. Lee SJ, Lee JH, Gillen M, Krause N. Job stress and work-related musculoskeletal symptoms among intensive care unit nurses: a comparison between job demand-control and effort-reward imbalance models. *Am J Ind Med*. (2014) 57:214-221. doi: 10.1002/ajim.22274.
16. Liu H, Li G, Zhang Z, Mao Y. Investigation of nurse work stress in grade 3 hospitals from different regions in China. *Med and Society*. (2013) 26:94-6. doi: 10.3870/YXYSH.2013.05.032.
17. Zhu Y, Liu J, Zhang Y, Liu C, Li M, Fan G. Influence of effort-reward imbalance of clinic nurses in pediatric department on turnover intention. *Chin Nurs Res*. (2013) 27:2728-30. doi: 10.3969/j.issn.1009-6493.2013.25.016.
18. Li J, Shang L, Galatsch M, Siegrist J, Miüller BH, Hasselhorn HM. Psychosocial work environment and intention to leave the nursing profession: a cross-national prospective study of eight countries. *Int J Health Serv*. (2013) 43:519-536. doi: 10.2190/HS.43.3.i.
19. Fang L, Hung C. Predictors of married female nurses' health. *Workplace Health Saf*. (2014) 62:447-55. doi: 10.3928/21650799-20140804-06.
20. Nourry N, Luc A, Lefebvre F, Sultan-Taïeb H, Béjean S. Psychosocial and organizational work environment of nurse managers and self-reported depressive symptoms: cross-sectional analysis from a cohort of nurse managers. *Int J Occup Med Environ Health*. (2014) 27:252-269. doi: 10.2478/s13382-014-0264-x.
21. Liu K, Zhang J, Zhou L. Influence of effort-reward imbalance on quality of nursing work life. *J Nurs Sci*. (2014) 29:52-5. doi: 10.3870/hlxzz.2014.07.052.
22. Yokoyama K, Hirao T, Yoda T, Yoshioka A, Shirakami G. Effort-reward imbalance and low back pain among eldercare workers in nursing homes: a cross-sectional study in Kagawa Prefecture, Japan. *J Occup Health*. (2014) 56:197-204. doi: 10.1539/joh.13-0295-oa.
23. Yuan C, Wei W. The current situation of effort-reward imbalance among nurses in Zhengzhou City and its impact on quality of life. *Health Vocat Educ*. (2015) 33:126-9.
24. Lin P, Chen C, Pan S, Chen Y, Pan C, Hung H, Wu M. The association between rotating shift work and increased occupational stress in nurses. *J Occup Health*. (2015) 57:307-15. doi: 10.1539/joh.13-0284-OA.
25. [Julia Claire](https://escholarship.org/search/?q=author:Buss, Julia Claire) B. Hospital work, occupational stress, and obesity among female registered nurses. *UC San Francisco Electronic Theses and Dissertations*. (2015).
26. Shi L. The research on the effects of occupational stress on quality of life in Surgical medical staff. *Xinjiang medical university*. (2015). MA thesis.
27. He X, Hao C. The relationship between occupational stress source,job burnout and depression of the nurses from community hospital. *Ind Health Occup Dis*. (2015) 41:250-3. doi: 10.13692/j.cnki.gywsyzyb.2015.04.003.
28. Lu Y. A Study on the strategy of relieving occupational stress and anxiety of nurses in JC Community Health Service Center. *East China University of Science and Technology*. (2016). MA thesis.
29. Martinez MC, Latorre MDRDO, Fischer FM. Stressors influence work ability in different age groups of nursing professionals: 2-year follow-up. *Cien Saude Colet*. (2017) 22:1589-1600. doi: 10.1590/1413-81232017225.09682015.
30. Chen T, Wong W, Huang W, Sun H. Factors associated with occupational tension and sense of effort-reward imbalance among nurses in grade A tertiary hospital. *Chin Ment Health J*. (2017) 31:862-4. doi: 10.3969/j.issn.1000-6729.2017.11.006.
31. Wang Y. A Study on the relationship between psychological capital, effort-reward imbalance and stay intention of pediatric nurses. *Henan university*. (2017). MA thesis.
32. Du J. Study on the effect of effort-reward imbalance and work adjustment disorder on the turnover intention of clinical nurses. *Jilin university*. (2017). MA thesis.
33. de Oliveira DR, Griep RH, Portela LF, Rotenberg L. Intention to leave profession, psychosocial environment and self-rated health among registered nurses from large hospitals in Brazil: a cross-sectional study. *BMC Health Serv Res*. (2017) 17:21. doi: 10.1186/s12913-016-1949-6.
34. Liu B, Jiang H, Qi Y, Zhang J, Wang Q. Study on the influencing factors of turnover intention among the nurses of the second grade hospitals in Beijing. *J Nurs Admin*. (2017) 17:90.
35. Wang J. A study on the correlation between psychological resilience and occupational stress and ability of nurses in military hospital. *Hunan normal university*. (2017). MA thesis. doi: 10.27137/d.cnki.ghusu.2017.000546.
36. Pinhatti EDG, Ribeiro RP, Soares MH, Martins JT, Lacerda MR, Galdino MJQ. Psychosocial aspects of work and minor psychic disorders in nursing: use of combined models. *Rev Lat Am Enfermagem*. (2018) 26:e3068. doi: 10.1590/1518-8345.2769.3068.
37. Li X, Lu Q, Wang L. Relationships among work stress, burnout and organizational commitment in operating room clinical nurse specialists employed in tertiary hospitals. *J Nurs Sci*. (2018) 33:1-4. doi: 10.3870/j.issn.1001-4152.2018.13.001.
38. Zaree TY, Nazari J, Asghary Jafarabadi M, Alinia T. Impact of Psychosocial Factors on Occurrence of Medication Errors among Tehran Public Hospitals Nurses by Evaluating the Balance between Effort and Reward. *Saf Health Work*. (2018) 9:447-453. doi: 10.1016/j.shaw.2017.12.005.
39. Liang J, Zhang J, Chen S. Correlation between effort-reward imbalance and career success in nurses of Class lll Grade A hospitals. *Chin J Mod Nurs*. (2018) 24:3864-7. doi: 10.3760/cma.j.issn.1674-2907.2018.32.005.
40. Colindres CV, Bryce E, Coral-Rosero P, Ramos-Soto RM, Bonilla F, Yassi A. Effect of effort-reward imbalance and burnout on infection control among Ecuadorian nurses. *Int Nurs Rev*. (2018) 65:190-199. doi: 10.1111/inr.12409.
41. Lua I, de Araújo TM, Santos KOB, de Almeida MMG. Factors associated with common mental disorders among female nursing professionals in primary health care. *Psicol Reflex Crit*. (2018) 31:20. doi: 10.1186/s41155-018-0101-4.
42. Deng X, Wei C, Xie Y, Li X, Tang S. Correlation investigation on effort-reward imbalance and organizational justice perception among nurses in tertiary hospital. *Occup Health*. (2018) 34:1797-99+1803. doi: 10.13329/j.cnki.zyyjk.2018.0497.
43. Salem EA, Ebrahem SM. Psychosocial work environment and oxidative stress among nurses'. *J Occup Health*. (2018) 60:182-191. doi: 10.1539/joh.17-0186-OA.
44. Fang L, Hsiao L, Fang S, Chen B. Associations of work stress and humor with health status in hospital nurses-A cross-sectional study. *J Clin Nurs*. (2019) 28:3691-99. doi: 10.1111/jocn.14970.
45. Chai Y, Jia Y, Pei L, Jia Y, Zhu C. The relationship between occupational stress and implicit absenteeism among emergency nurses. *Chin J Emerg Crit Care Nurs*. (2020) 1:389-94. doi: 10.3761/j.issn.2096-7446.2020.05.001.
46. Kong L, Li W, Wang H, Xu N, Xu Q, Sun L. The relationship between effort-reward imbalance and empathy among clinical nurses: A cross-sectional online survey. *J Clin Nurs*. (2020) 29:3363-72. doi: 10.1111/jocn.15367.
47. Bardhan R, Heaton K, Davis M, Chen P, Dickinson DA, Lungu CT. A Cross Sectional Study Evaluating Psychosocial Job Stress and Health Risk in Emergency Department Nurses. *Int J Environ Res Public Health*. (2019) 16:3243. doi: 10.3390/ijerph16183243.
48. Kabakleh Y, Zhang J, Lv M, Li J, Yang S, Swai J. Burnout and associated occupational stresses among Chinese nurses: A cross-sectional study in three hospitals.*PLoS One*. (2020) 15:e0238699. doi: 10.1371/journal.pone.0238699.
49. Wang J, Sang S, Zhang J. The effects of psychological capital and effort-reward imbalance on ICU nurses’ turnover intention. *Guangxi Med J*. (2020) 42:2178-82+86. doi: 10.11675/j.issn.0253-4304.2020.16.31.
50. Babazadeh M, Molavynejad S, Parhamnia Z, Boroun T. Interplay of occupational stress, sense of humor, and health status among nurses working at hospitals in Ahvaz. *J Med Life*. (2021) 14:262-270. doi: 10.25122/jml-2020-0032.
51. Gao Y. Investigation on occupational stress and its related factors of medical staff in a third-grade general hospital in Hebei province. *North China University of Science and Technology*. (2021). MA thesis. doi: 10.27108/d.cnki.ghelu.2021.000030.
52. Su M, Zhang S, Chen Y, Li L, Wang J. Correlation between occupational stress and safe behavior of new nurses in standardized training in operating room. *Chin J Health Psychol*. (2021) 29:1854-8. doi: 10.13342/j.cnki.cjhp.2021.12.021.
53. Tzenetidis V, Papathanasiou I, Tzenetidis N, Nikolentzos A, Sarafis P, Malliarou M. Effort Reward Imbalance and Insomnia Among Greek Healthcare Personnel During the Outbreak of COVID-19. *Mater Sociomed*. (2021) 33:124-130. doi: 10.5455/msm.2021.33.124-130.
54. Tian M, Yang H, Yin X, Wu Y, Zhang G, Lv C. Evaluating effort-reward imbalance among nurses in emergency departments: a cross-sectional study in China. *BMC Psychiatry.* (2021) 21:353. doi: 10.1186/s12888-021-03344-6.
55. Li J. Latent profile analysis on job burnout and related cumulative risk among psychiatric nurses. *Shandong University*. (2021). MA thesis. doi: 10.27272/d.cnki.gshdu.2021.005509.
56. Martinez MC, Latorre MDRDO, Fischer FM. Factors associated with work ability and intention to leave nursing profession: a nested case-control study.*Ind Health*. (2022) 60:29-39. doi: 10.2486/indhealth.2021-0085.
57. Gao X, Wang Z, Ai Y, Xu M, Zheng S. Cross-sectional survey on occupational stress and job burnout in female nurses in tertiary general hospitals of Wuhan city. *Occup Health*. (2022) 38:1881-6. doi: 10.13329/j.cnki.zyyjk.2022.0444.
58. Yan J, Wu C, Du Y, He S, Shang L, Lang H. Occupational Stress and the Quality of Life of Nurses in Infectious Disease Departments in China: The Mediating Role of Psychological Resilience. *Front Psychol*. (2022) 13:817639. doi: 10.3389/fpsyg.2022.817639.
59. Gustavsson K, Jernajczyk W, Wichniak A. Insomnia partially mediates the relationship of occupational stress with mental health among shift working nurses and midwives in polish hospitals. *Nat Sci Sleep*. (2022) 14:1989-1999. doi: 10.2147/NSS.S375375.
60. An R, Dong Y, Yan Q, Zhu L, Li Q, Yan S, et al. Effort-reward imbalance and related factors among nurses in emergency department of central China. *Chin Ment Health J*. (2023) 37:429-34. doi: 10.3969/j.issn.1000-6729.2023.05.012.

**PRISMA_2020_checklist**

| **Section and Topic** | **Item #** | **Checklist item** | **Location where item is reported** |
| --- | --- | --- | --- |
| **TITLE** | | |  |
| Title | 1 | Identify the report as a systematic review. | See the Title. |
| **ABSTRACT** | | |  |
| Abstract | 2 | See the PRISMA 2020 for Abstracts checklist. | See the Abstract. |
| **INTRODUCTION** | | |  |
| Rationale | 3 | Describe the rationale for the review in the context of existing knowledge. | See the Introduction，the Paragraph 4. |
| Objectives | 4 | Provide an explicit statement of the objective(s) or question(s) the review addresses. | See the Introduction，the Paragraph 4. |
| **METHODS** | | |  |
| Eligibility criteria | 5 | Specify the inclusion and exclusion criteria for the review and how studies were grouped for the syntheses. | See the Methods, “Search strategy”. |
| Information sources | 6 | Specify all databases, registers, websites, organisations, reference lists and other sources searched or consulted to identify studies. Specify the date when each source was last searched or consulted. | See the Methods, “Study selection”. |
| Search strategy | 7 | Present the full search strategies for all databases, registers and websites, including any filters and limits used. | See the Methods, “Study selection”. |
| Selection process | 8 | Specify the methods used to decide whether a study met the inclusion criteria of the review, including how many reviewers screened each record and each report retrieved, whether they worked independently, and if applicable, details of automation tools used in the process. | See the Methods, “Study screening and data extraction”, the paragraph 1. |
| Data collection process | 9 | Specify the methods used to collect data from reports, including how many reviewers collected data from each report, whether they worked independently, any processes for obtaining or confirming data from study investigators, and if applicable, details of automation tools used in the process. | See the Methods, “Study screening and data extraction”, the paragraph 1. |
| Data items | 10a | List and define all outcomes for which data were sought. Specify whether all results that were compatible with each outcome domain in each study were sought (e.g. for all measures, time points, analyses), and if not, the methods used to decide which results to collect. | See the Methods, “Study screening and data extraction”, the paragraph 2. |
|  | 10b | List and define all other variables for which data were sought (e.g. participant and intervention characteristics, funding sources). Describe any assumptions made about any missing or unclear information. | See the Methods, “Study screening and data extraction”, the paragraph 2. |
| Study risk of bias assessment | 11 | Specify the methods used to assess risk of bias in the included studies, including details of the tool(s) used, how many reviewers assessed each study and whether they worked independently, and if applicable, details of automation tools used in the process. | See the Methods, “Quality assessment”. |
| Effect measures | 12 | Specify for each outcome the effect measure(s) (e.g. risk ratio, mean difference) used in the synthesis or presentation of results. | See the Methods, “Statistical analysis”. |
| Synthesis methods | 13a | Describe the processes used to decide which studies were eligible for each synthesis (e.g. tabulating the study intervention characteristics and comparing against the planned groups for each synthesis (item #5)). | See the Methods, “Statistical analysis”. |
|  | 13b | Describe any methods required to prepare the data for presentation or synthesis, such as handling of missing summary statistics, or data conversions. | See the Methods, “Statistical analysis”. |
|  | 13c | Describe any methods used to tabulate or visually display results of individual studies and syntheses. | See the Methods, “Statistical analysis”. |
|  | 13d | Describe any methods used to synthesize results and provide a rationale for the choice(s). If meta-analysis was performed, describe the model(s), method(s) to identify the presence and extent of statistical heterogeneity, and software package(s) used. | See the Methods, “Statistical analysis”. |
|  | 13e | Describe any methods used to explore possible causes of heterogeneity among study results (e.g. subgroup analysis, meta-regression). | See the Methods, “Statistical analysis”. |
|  | 13f | Describe any sensitivity analyses conducted to assess robustness of the synthesized results. | See the Methods, “Statistical analysis”. |
| Reporting bias assessment | 14 | Describe any methods used to assess risk of bias due to missing results in a synthesis (arising from reporting biases). | See the Methods, “Statistical analysis”. |
| Certainty assessment | 15 | Describe any methods used to assess certainty (or confidence) in the body of evidence for an outcome. | See the Methods, “Statistical analysis”. |
| **RESULTS** | | |  |
| Study selection | 16a | Describe the results of the search and selection process, from the number of records identified in the search to the number of studies included in the review, ideally using a flow diagram. | See the Results, “Selection of studies and basic characteristics” paragraph 1 and Figure 1. |
|  | 16b | Cite studies that might appear to meet the inclusion criteria, but which were excluded, and explain why they were excluded. | See the Results, “Selection of studies and basic characteristics” paragraph 1 and Figure 1. |
| Study characteristics | 17 | Cite each included study and present its characteristics. | See the Results, “Selection of studies and basic characteristics” paragraph 2 and Table 1. |
| Risk of bias in studies | 18 | Present assessments of risk of bias for each included study. | See the Results, “Quality assessment” and Supplementary material 1. |
| Results of individual studies | 19 | For all outcomes, present, for each study: (a) summary statistics for each group (where appropriate) and (b) an effect estimate and its precision (e.g. confidence/credible interval), ideally using structured tables or plots. | See the Results, “Selection of studies and basic characteristics” and Table 1. |
| Results of syntheses | 20a | For each synthesis, briefly summarise the characteristics and risk of bias among contributing studies. | See the Results, “Incidence of effort-reward imbalance in Chinese nurses” and Figure 1. |
|  | 20b | Present results of all statistical syntheses conducted. If meta-analysis was done, present for each the summary estimate and its precision (e.g. confidence/credible interval) and measures of statistical heterogeneity. If comparing groups, describe the direction of the effect. | See the Results, “Subgroup analysis” and Table 2. |
|  | 20c | Present results of all investigations of possible causes of heterogeneity among study results. | See the Results, ”Publication bias and sensitivity analysis” and “Subgroup analysis”. |
|  | 20d | Present results of all sensitivity analyses conducted to assess the robustness of the synthesized results. | See the Results, ”Publication bias and sensitivity analysis”, the paragraph 2. |
| Reporting biases | 21 | Present assessments of risk of bias due to missing results (arising from reporting biases) for each synthesis assessed. | See the Results, ”Publication bias and sensitivity analysis”, the paragraph 1. |
| Certainty of evidence | 22 | Present assessments of certainty (or confidence) in the body of evidence for each outcome assessed. | See Table 1 and Table 2, Figure 1, Figure2, Figure3 and Figure4. |
| **DISCUSSION** | | |  |
| Discussion | 23a | Provide a general interpretation of the results in the context of other evidence. | See the Discussion, the Paragraph 1~6. |
|  | 23b | Discuss any limitations of the evidence included in the review. | See the Discussion, the Paragraph 7. |
|  | 23c | Discuss any limitations of the review processes used. | See the Discussion, the Paragraph 7. |
|  | 23d | Discuss implications of the results for practice, policy, and future research. | See the Discussion, “Conclusion”. |
| **OTHER INFORMATION** | | |  |
| Registration and protocol | 24a | Provide registration information for the review, including register name and registration number, or state that the review was not registered. | See the Method, “Protocol”. |
|  | 24b | Indicate where the review protocol can be accessed, or state that a protocol was not prepared. | See the Method, “Protocol”. |
|  | 24c | Describe and explain any amendments to information provided at registration or in the protocol. | See the Method, “Protocol”. |
| Support | 25 | Describe sources of financial or non-financial support for the review, and the role of the funders or sponsors in the review. | See the “Funding” and “Acknowledgments”. |
| Competing interests | 26 | Declare any competing interests of review authors. | See the “Disclosure”. |
| Availability of data, code and other materials | 27 | Report which of the following are publicly available and where they can be found: template data collection forms; data extracted from included studies; data used for all analyses; analytic code; any other materials used in the review. | All the data can be found in the Supplementary materials, which has been mentioned in the manuscript. |

*From:*  Page MJ, McKenzie JE, Bossuyt PM, et al. The PRISMA 2020 statement: an updated guideline for reporting systematic reviews. BMJ. 2021;372:n71. doi: 10.1136/bmj.n71 For more information, visit: <http://www.prisma-statement.org/>A
